# A mediated moderation model of negative life events, self-esteem, rumination and parental divorce on depression among Chinese juvenile delinquents

**DOI:** 10.1038/s41598-023-28626-9

**Published:** 2023-01-31

**Authors:** Shou-Ying Zhao, Rong-Rong Ren, Wei Chen

**Affiliations:** 1 School of Psychology, Guizhou Normal University, Guiyang, Guizhou China; 2 Kaili University, Kaili, Guizhou China

**Keywords:** Psychology, Human behaviour

## Abstract

Little attention was paid to the prevalence of depression in Chinese juvenile delinquents who are studied in correctional work-study schools. Hence, the present study aimed to test the unique, mediating and moderating effects of negative life events, self-esteem, rumination and parental divorce on depression among Chinese juvenile delinquents. A total of 236 juvenile delinquents aged between 12 and 17 years old (M = 14.68 SD = 1.30) were recruited to accomplish a battery of self-report questionnaires concerning their negative life events, self-esteem, rumination, depression and demographic profile. The descriptive analysis showed that there was a positive correlation between negative life events and depression in Chinese juvenile delinquents. Moreover, the results from the structural model indicated that self-esteem and rumination played separate and sequential mediating roles between negative life events and depression. In addition, parental divorce had a moderating effect between negative life events and self-esteem in the model. These results suggest that the prevalence of depression among Chinese juvenile delinquents can be reduced through modification of the ruminative way of thinking, improving their self-esteem when they encounter a number of negative life events. Furthermore, more attention should be paid to the juvenile delinquents who experienced parental divorce.

## Introduction

Depression is one of the most common mental disorders among children and adolescents and it may lead to serious psychological distresses^1,2^. A nationally representative survey of U.S. indicated that the depression rates among adolescents increased 52%, from 8.7 to 13.2% 2005–2017^3^. Moreover, a meta-regression analysis, involving 33 studies with 18,861 detained adolescents, showed that 10.1% male adolescents and 25.8% female adolescents had depression^4^. Therefore, the detained adolescents are a group that deserves to be focused on, but there are numerous researchers who pay close attention to community students’ depression^5,6^. Although some western studies are concerned about the depression of adolescents who are in juvenile detention and correctional facilities, the studies upon Chinese juvenile delinquents’ depression are scarce^4^. Hoare et al. considered that at an individual level, the drivers of depression would be different and therefore, the effectiveness of preventive interventions would be also likely to vary^7^. That is, the prevention may be efficient among people without offending behavior, but it may not be effective for juvenile delinquents. Therefore, the present research addresses this gap by exploring these factors (e.g., negative life events, self-esteem, rumination and parental divorce) that may make a contribution to decrease or increase the level of depression in juvenile delinquents.

Juvenile delinquents here are the students who study in correctional work-study schools for they have serious juvenile misbehaviors, and their illegal act is not serious enough to put them into jail^8^. Although some researchers claimed that juvenile delinquents’ depression level was significantly higher than that of community students and they had more mental problems^9^, there were few researches on the magnitude of depression and the mechanisms among these high-risk students in China. However, depression in this group is connected with a range of negative outcomes, including the increasing risk of being recidivists^10^ and great odds of having substance use disorders^11^. In respect of prevention programs, it is essential to identify risk and protective factors before the development of depression. Hence, it is imperative to establish how some factors exert its effect on Chinese juvenile delinquents’ depression.

Negative life events often refer to as adverse events, stressful events, stressors, chronic events, or traumas^12^. Many researchers confirmed that the relationship between experiences of adverse life events and depression among adolescents was significant^13^. A longitudinal study found that negative life events might result in depression regardless of the children’s age^14^. Moreover, in the light of general strain theory, stressful life events would result in negative emotions, which in turn, increased the probability of adolescents’ involvement in criminal behavior^15^. Beck put forward the cognitive model of depression and concluded that early life stress sensitized individuals to later negative life events through impact on cognitive vulnerability leading to depression^16^. Furthermore, it was found that stressful life events predicted depressive symptoms one year later in an adolescent sample^17^.

From the above analyses, it can be seen that negative life events, as external stressor, have an impact on juvenile delinquents’ depression. One study found that depression is not only affected by external factors (e.g., negative life events) but also affected by internal factors (e.g., self-esteem, psychological resilience)^18^. External factors are often active through internal factors, so it is necessary to investigate the underlying mechanisms between negative life events and depression among the high-risk group.

According to previous researches, self-esteem is seemed to be a mediator between negative life events and depression, for it strongly correlates with the two variables^19,20^. Rosenberg et al. stated that self-esteem refers to the individual’s positive or negative attitude toward the self as a totality^21^, and some researchers found that high self-esteem played a protective role in helping adolescents who suffered from depression^22^. Moreover, some studies supported the vulnerability model, which stated that low self-esteem was considered as a potential risk factor for depression^23^. Furthermore, one study demonstrated that the prospective effect of stressful life events on depression became weaker as self-esteem increased^24^. Previous studies indicated that low self-esteem might result in delinquency^25^, in addition, self-esteem had been known to be less stable during adolescence after which it became relatively stable across one’s lifetime^26^. Therefore, it is imperative to clarify the effect of self-esteem on depression among juvenile delinquents who tend to have a relatively high ratio of exposure to negative life events.

Even there are so many studies on the association between self-esteem and depression, but few lays their emphasis on the question of whether there is any mediating mechanism connecting the two variables. Kuster et al. found that rumination partially mediated the relationship between low self-esteem and depression^23^. Moreover, rumination played a mediating role in the association between stressful life events and major depression among diagnosed individuals and healthy individuals^27^. Therefore, rumination is seemed to be a mediator among negative life events, self-esteem and depression. Rumination is defined as a pattern of repetitive thinking about one’s negative affect and is hypothesized to be considered as a susceptibility factor in the development and maintenance of depressive symptoms^28^. The relationship between rumination—considered as a negative cognitive style—and depression had been widely examined over the past decade. Robinson and Alloy proposed a conceptual extension of the response styles theory^29^ and hypothesized that individuals who exhibited an inclination to ruminate on these negative cognitions in response to stressful life events should be more prone to experience onsets of depression first^30^. Furthermore, rumination had been identified as a risk factor for the development of depressive symptoms when adolescents experienced negative life events^31^. In addition, one study stated that one of the reasons why low self-esteem might increase rumination was that people whose self-esteem was persistently low might cause negative self-related thoughts to enter their focus of attention repeatedly by rumination^23^. Therefore, according to the above analyses, rumination is not only a cognitive variable between negative life events and depression but also is considered as a possible mediator between self-esteem and depression.

Among juvenile delinquents, their depression level varies from person to person, even if they experienced the same number of negative life events. That is, there exists some protective factors that can reduce the risk of developing depression, which indicates that some moderators are between negative life events and depression. Aro found that the scarce of family cohesion and marital discord would be a vulnerability factor in depression^32^. Hence, parental divorce may be a possible moderator. One meta-analysis suggested that parental divorce was one of the most crucial negative life events in one’s childhood^33^. And, divorce can bring about the parent–child relation with interruption, economic distress and an increase in the number of other negative life events^34^. It is found that individuals whose parents are divorced have a higher risk of depression than those who are from nondivorced families^35^. Therefore, parental divorce would be a potential moderator between negative life events and depression. In addition, divorce-related stigma still exists in most rural areas in China^36^. Not only cross-sectional studies showed that there was a relationship between stigma and low self-esteem, but also a longitudinal study indicated that stigma played a casual role in reducing the self-esteem level^37^. Hence, the present study assumes that parental divorce plays a moderated role between negative life events and self-esteem.

### The present study

**Figure 1:**
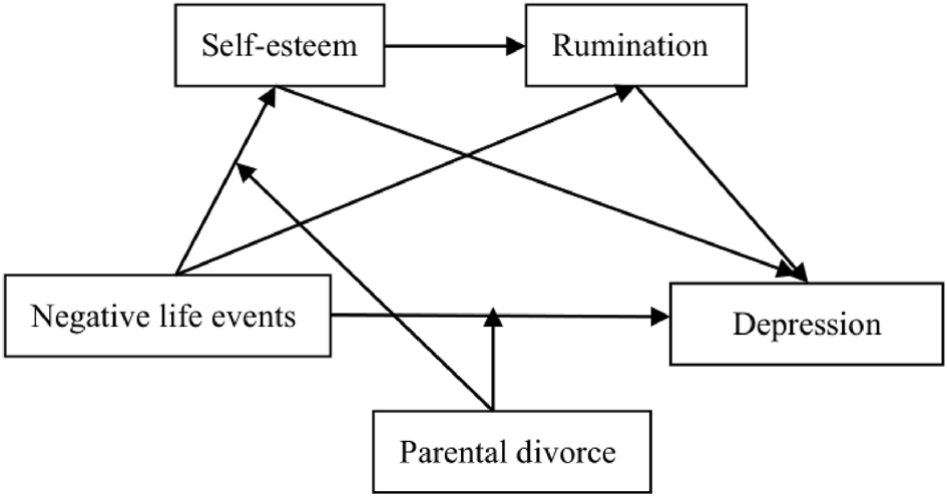
The proposed moderated mediation model.

The primary goal of the current study is to extend previous research with regard to the relationship between negative life events and depression among Chinese juvenile delinquents and to test the mechanisms behind this association. According to the previous studies, four main hypotheses are tested. First, we hypothesize that negative life events have a positive relationship with depression. Next, we hypothesize that the association between negative life events and depression is mediated by self-esteem and rumination. Then, we hypothesize that self-esteem and rumination serially mediate the relationship between negative life events and depression. Last, we hypothesize that not only the association between negative life events and depression but also the association between negative life events and self-esteem are moderated by parental divorce. The proposed model is illustrated in Fig. 1.

## Methods

### Participants and procedure

249 juvenile delinquents were recruited from correctional work-study schools from one province in China. Among them, 8 participants whose missing answers of a scale less than 50% were excluded and the data of 5 participants was deleted for their answers were all the same option no matter the items were. Most of the participants completed the instruments without any omission and the missing rate of the remained data was less than 20% each scale. EM algorithm was used to impute the missing data. Eventually, the study group consisted of a total of 236 juvenile delinquents. All delinquents here had serious misconduct behaviors that referred to the illegal behavior that seriously endangered the society but were not enough for criminal punishment. Moreover, the delinquents in work-study schools can get their education as well. The subjects’ age ranged from 12 to 17, with a mean age of 14.68 years (SD = 1.30). For the total sample, 206 were boys (87.3%) and 30 were girls (12.7%); 65.3% were from rural areas and 34.7% were from town. The duration of schooling, which refers to the time they stayed in the correctional school, was varied from different groups: 3–6 months (21.2%), 6–12 months (53.4%) and more than one year (25.4%). Among these participants, 95 (40.3%) juvenile delinquents whose parents were divorced and the only child accounted for 21.2%.

All research methods were carried out in accordance with relevant ethical guidelines and regulations. Informed consent was obtained from all participants. Besides, participants who were younger than 16 years should get their legal guardians’ informed consent. The study was approved by the Guizhou Normal University Human Research Ethics Committee. We contacted with the director in advance and chose the time that was convenient for the participants to complete the scales in a classroom. Before administering the questionaries, our team members, well-trained psychology postgraduate students, would explain the purposes of the study to participants and inform that they had the right to withdraw at any time. Moreover, complete anonymity was guaranteed to respondents. Then, each participant was received a paper-and-pencil questionnaire and it would took them approximately twenty minutes to complete.

### Measures

#### Demographic questionnaire

Before accomplishing the scales, participants filled out a demographic questionnaire concerning their gender (boy = 1, girl = 2), place of residence (rural = 1, town = 2), only child or not (yes = 1, no = 2), parental divorce or not (yes = 1, no = 0) and duration of schooling, which refers the time they studied in correctional work-study school (3 months = 1, 6 months = 2, one year = 3).

#### Negative life events

The Adolescent Self-rating Life Events Check-list (ASLEC) is a 27-item self-report scale, which was used to assess the impact of negative life events that happened to the participants in the past 12 months^38^. The checklist comprised of 6 subscales including interpersonal relationships (e.g., I was misunderstood), academic pressure (e.g., I failed in the examination), being punished (e.g., I was criticized in school), loss (e.g., I was seriously ill), health and adaptation problem (e.g., I was away from family) and others (e.g., I hated going to school). Participants were asked to answer the items whether they experienced these events. If the event happened to them, they had to rate items with a five-point Likert scale to assess the impact of the events on them (1 = no impact; 5 = extremely severe impact). Otherwise, they only chose the option ‘not applicable’ and we would assign zero to this option. A sum score was used by adding up all the subscales score together. A higher score suggested a greater impact of negative life events. The reliability of the original scale was 0.849 and the scale in the current study demonstrated a good reliability (α = 0.809).

#### Rumination

We adopted the Chinese version of the Ruminative Responses Scale (RRS) to measure rumination^29,39^. The RRS scale was made up of 22 items divided into three subscales that were symptom rumination (e.g., Think “I feel lonely”), brooding (e.g., Think “Why do I always react this way?”) and reflective pondering (e.g., Go someplace alone to think of my feeling). Each item was rated on a Likert scale ranging from 1 (almost never) to 4 (almost always). The total score of rumination was computed by adding all the items together, with a higher score indicating a higher level of rumination. The internal consistency of the original scale was 0.900 and the test–retest reliability was 0.820. In the present study, the Cronbach α coefficient for the scale was 0.866.

#### Self-Esteem

The variable self-esteem was assessed by means of the Chinese adaption of the Rosenberg Self-Esteem Scale (RSES) ^40^. The RSES includes 10 items rated on a 4-point scale ranging from 1 (strongly disagree) to 4 (strongly agree) and item 3, 5, 8, 9 and 10 were reverse-coded. Higher scores on the RSES represented greater levels of self-esteem. The RSES was used in a large sample involved middle and high school students in China and its Cronbach α coefficient was 0.870^41^. The internal consistency in the current sample was acceptable (α = 0.717).

#### Depression

The Chinese version of the Center for Epidemiologic Studies Depression Scale (CES-D) was a widely used measure for nonclinical people in China^42^. It included 20 items comprising four subscales reflecting depression: depressed affect (e.g., I felt depressed), positive affect (e.g., I felt that I was just as good as other people), somatic and retarded activity (e.g., I had trouble keeping my mind on what I was doing) and interpersonal relationship (e.g., People were unfriendly). Items were scored from 1 = occasionally to 4 = always; the total score ranged from 20 to 80, with a cutoff point of 36 or above suggesting depression, and individuals with higher scores would suffer from severer depression. The reliability of the original scale was 0.900 and the CES-D scale in the current study was considered good (α = 0.807).

### Statistical analysis

In the descriptive statistics, we calculated the means, standard deviations, and Pearson bivariate correlations among main variables using SPSS 22.0. And, considering that the SPSS macro PROCESS is specifically developed for testing complex models including both mediator and moderator variables, the serial mediation model and the mediated moderation model in the current study were conducted using SPSS 22.0 including a Process macro version 3.0^43^^.^ The first model aimed to testify the indirect effects of negative life events on depression through the mediating roles of self-esteem and rumination. Then we examined the moderating effect of parental divorce on the association between negative life events and self-esteem and the relationship between negative life events and depression in the multiple mediation model. The bootstrapping resampling method was used to test the significance of indirect effects with 5000 subsamples to estimate. The present study utilized the 95% confidence interval of the indirect effects, and the effects are significant at α = 0.05 when the confidence intervals for the parameter estimate do not contain zero^44^. The main variables in the serial mediation model and the mediated moderation model were standardized and the analyses were all based on Z-scores.

## Results

### Descriptive analysis

The descriptive statistics and the Pearson bivariate correlations among variables were displayed in Table 1. As shown in Table 1, among the demographic variables, only the duration of schooling was significantly related with depression. Juvenile delinquents who studied in the correctional work-study school for a longer time were more likely to have a low level of depression. Hence, the study time in correctional work-study school was controlled for in the serial mediation analysis and the moderation analysis. We found that there were significant intercorrelations among negative life events, self-esteem, rumination and depression.

**Table 1:** Means, Standard Deviations, Range of the study variables and Bivariate Correlations among them.

Variables	Mean	SD	Range	Correlations with self-esteem	Correlations with rumination	Correlations with depression
Gender	–	–	–	− 0.125	0.110	0.121
Place of residence	–	–	–	0.006	− 0.093	− 0.062
Only child or not	–	–	–	0.018	− 0.018	− 0.029
Duration of schooling	–	–	–	0.109	− 0.094	− 0.178**
Self-esteem	26.123	4.164	16–38	–	− 0.285***	− 0.523***
Rumination	47.670	9.819	27–79	–	–	0.469***
Depression	40.060	9.002	21–73	–	–	–
Negative life events	61.589	15.090	26–107	− 0.219**	0.404***	0.296***
Interpersonal relationships	11.585	3.440	5–23	− 0.246***	0.424***	0.355***
Academic pressure	10.936	3.814	3–22	− 0.196**	0.327***	0.259***
Being punished	15.631	5.194	5–35	− 0.103	0.213**	0.093
Loss	5.297	2.602	0–13	0.019	0.221**	0.188**
Health and adaption problem	8.220	2.759	0–18	− 0.208**	0.296***	0.335***
others	9.920	3.435	3–20	− 0.189**	0.258***	0.108

N = 236. Duration of schooling means that the time the participants studied in the correctional work-study school. ***p < 0.001, **p < 0.01, *p < 0.05.

### The mediating roles of self-esteem and rumination

**Figure 2:**
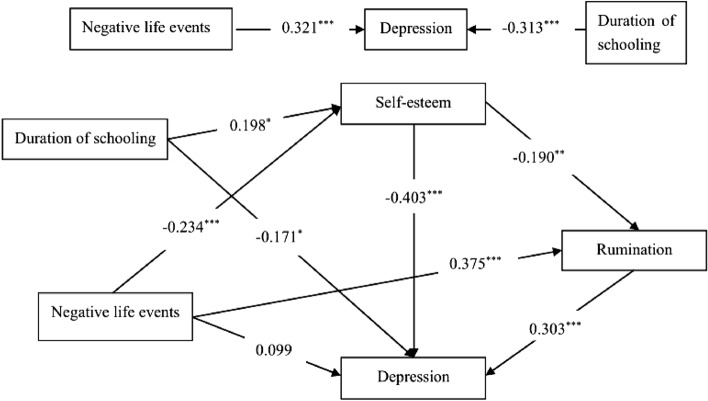
The mediating roles of self-esteem and rumination between negative life events and depression. Duration of schooling is treated as a controlled variable.

This study selected Model 6 in PROCESS macro to analyze the serial multiple mediator model. In the model, the time the participants stayed in the correctional work-study school (duration of schooling) was treated as a covariate variable. As shown in Fig. 2 and Table 1, the total effect of negative life events on depression was significant (β = 0.321, *p* < 0.001), and the positive correlation between the two variables was significant (r = 0.296, *p* < 0.001). The first hypothesis was confirmed. In the serial multiply mediator model, the direct relationship between negative life events and depression was not significant (β = 0.099, *p* > 0.05). However, negative life events had indirect effects on depression mainly through three paths. First, negative life events predicted self-esteem directly (β = − 0.234, *p* < 0.001) and self-esteem was negatively associated with depression (β = − 0.403, *p* < 0.001). Then, negative life events predicted rumination positively (β = 0.375, *p* < 0.001) and rumination positively related with depression (β = 0.303, *p* < 0.001). Last, self-esteem predicted rumination negatively and significantly (β = − 0.190, *p* < 0.01).

The mediating effect results were showed in Table 2. The 95% bootstrapped confidence interval of each indirect effect did not include zero value, which represented that the mediating effect was significantly different from zero. It indicated that all the mediating effects between negative life events and depression were significant; the total mediating effect was 0.221. The total indirect effect consisted of the following three paths: (1) Negative life events affected Chinese juvenile delinquents’ depression through self-esteem (β = 0.094), accounting for 42.5% of the total indirect effect, (2) Rumination played a mediating role between negative life events and depression among Chinese juvenile delinquents (β = 0.114), accounting for 51.6% of the total indirect effect, and (3) Negative life events affected Chinese juvenile delinquents’ depression through the chain mediating roles of self-esteem and rumination (β = 0.013), accounting for 5.9% of the total indirect effect. The above analyses supported the chain mediating effects of self-esteem and rumination on the relation between negative life events and depression.

**Table 2:** Summary of path coefficients and mediating effects tests.

Dependent variable	Model paths	Coefficient (β)	SE	Bootstrap 95%	
				LLCI	ULCI
Depression	**Total indirect effect:** Negative life events → Depression	0.221	0.043	0.139	0.307
	**Indirect effect(Path 1):** Negative life events → Self-esteem → Depression	0.094	0.028	0.042	0.150
	**Indirect effect(Path 2):** Negative life events → Rumination → Depression	0.114	0.032	0.055	0.182
	**Indirect effect(Path 3):** Negative life events → Rumination → Self-esteem → Depression	0.013	0.007	0.003	0.029

### The moderating role of parental divorce

**Figure 3:**
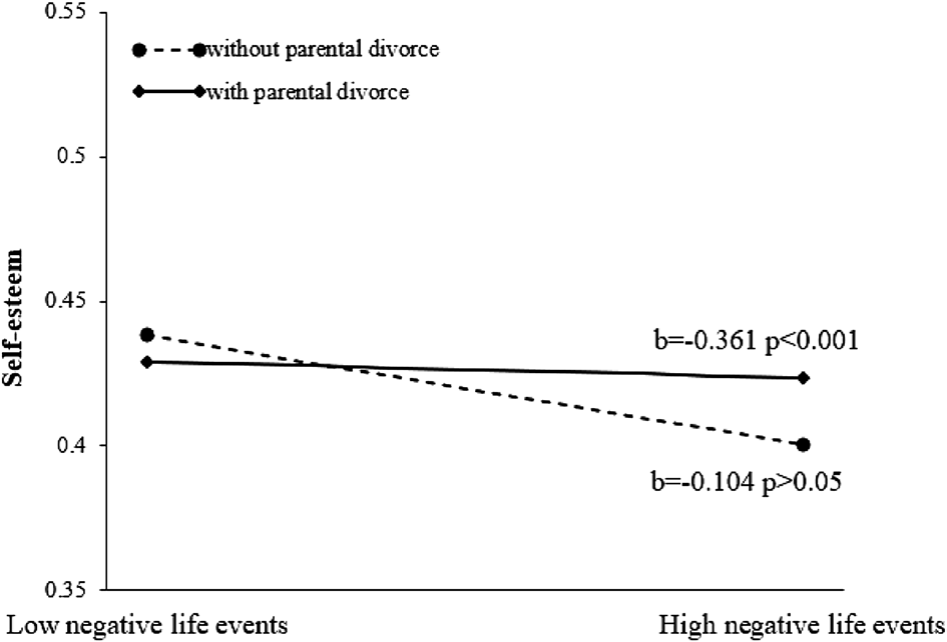
Self-esteem as a function of Negative life events and Parental divorce. Functions are graphed for two conditions: with parental divorce and without parental divorce.

The current study presumed that the moderating effect of parental divorce on the relationship between negative life events and self-esteem (Model 1) and the association between negative life events and depression (Model 2). The mediated moderation model here were assessed with Model 86 in PROCESS macro. The model coefficients were showed in Table 3. According to the results, there were no significant main effects of negative life events and parental divorce in predicting self-esteem. As expected, it showed that there was a statistically significant interaction effect of negative life events and parental divorce on self-esteem (β = − 0.257, *p* < 0.05) in Model 1. To further illustrate the Negative life events × Parental divorce interaction for self-esteem, we plotted a simple slope and decomposed the interaction by juvenile delinquents with and without parental divorce experience (see Fig. 3). As can be seen in Fig. 3, the results showed that negative life events and self-esteem had a significant relationship only among juvenile delinquents with parental divorce experience (β = − 0.361, *p* < 0.001), and not among those without parental divorce experience (β = − 0.104, *p* > 0.05). That is, high level of negative life events (1 SD above the mean) would have an adverse effect on self-esteem among juvenile delinquents with parental separation. Additionally, for juvenile delinquents with high level of negative life events (1 SD above the mean), those with an intact family would have a higher level of self-esteem than those with parental divorce. But in Model 2, only self-esteem and rumination had main effects on depression, and the effect of negative life events on depression was not significantly moderated by parental divorce (β = − 0.110, *p* = 0.291).

**Table 3:** Model coefficients for the conditional process model.

Antecedent	Consequent							
	Model 1 (Self-esteem)				Model 2 (Depression)			
	Coefficient	SE	t	*p*	Coefficient	SE	t	*p*
Negative life events	− 0.104	0.088	− 1.176	0.241	0.147	0.076	1.936	0.054
Parental divorce	− 0.112	0.130	− 0.868	0.387	0.081	0.105	0.769	0.443
Negative life events × Parental divorce	− 0.257	0.127	− 2.02	0.045	− 0.110	0.104	− 1.058	0.291
Self-esteem	–	–	–	–	− 0.408	0.054	− 7.501	< 0.001
Rumination	–	–	–	–	0.300	0.058	5.208	< 0.001
	R^2^ = 0.016 F = 4.078 *P* < 0.05				R^2^ = 0.003 F = 1.118 *P* > 0.05

## Discussion

The main purpose of this study was to reveal the underlying mechanism of the association between negative life events and depression among Chinese juvenile delinquents and then, four hypotheses were put forward. The first hypothesis was consistent with previous studies and the results showed that negative life events could positively relate with Chinese juvenile delinquents’ depression^13^. It suggested that exposure to negative life events could place juvenile delinquents at a risk for developing depression especially they always had a high probability to experience more negative life events than non-delinquent adolescents^45^. Moreover, the present study found that negative life events regarding interpersonal relationships, health and adaptation problems had a greater impact on juvenile delinquents’ depression than other negative life events.

Besides, the results showed that self-esteem and rumination played separate and sequential mediating roles between negative life events and depression. The results might provide a convincing evidence to the diathesis-stress model for depression. The diathesis-stress model is always used to explain the etiology of depression^46^ and the model suggests that the impacts on the depression risk rely on the diathesis or vulnerability^47^. Rick and David gave an explanation of a significant interaction that stress activates the diathesis, which in turn causes the onset of disorder^48^. Therefore, negative life events were seen as stress that had a significant total effect on depression, which confirmed the vital effect of stress on depression. In addition, the indirect effect of negative life events on depression through self-esteem and then rumination indicated that the combination of stress (negative life events) and diathesis (self-esteem and rumination) made contributions to depression together.

Moreover, in accordance with previous studies^24^, the mediated effect of self-esteem on the relation between negative life events and depression among juvenile delinquents was significant. That is, when individuals encounter a number of negative life events, those with high self-esteem will be less likely to be vulnerable to depression. But from the descriptive statistics, it has found that the mean score of self-esteem among juvenile delinquents is lower than community adolescents^49,50^, which is confirmed by the previous study^51^. Therefore, self-esteem might be a vital protective factor that can be targeted in the early intervention so as to reduce depression. Comparing with high self-esteem, youths’ self-concept and identities will be damaged by low self-esteem in the long-term, as well as the short-term, so that persons with low self-esteem are more likely to have trouble in believing people and obtaining less social support^52^. It happened to be found in our study that interpersonal relationship was the one among negative life events affected juvenile delinquents’ self-esteem most. Hence, low self-esteem with interpersonal difficulties should be in relation to increasing depression in middle adulthood^25^. Moreover, the study of Park and Yang found that self-esteem during young adulthood remained a determinant of high depression in middle adulthood^25^.

In addition, negative life events would influence depression through rumination among juvenile delinquents, indicating that rumination might also be a vital mechanism behind the relationship between negative life events and depression. In other words, it means that negative life events predict increases in depression when couple with higher levels of rumination. The results are aligned with other studies^31^. In line with ruminative response style^29^, youths who show a ruminative response style when they become depressed, focusing on negative aspects, are more tendency to aggravate temporary negative mood states. Therefore, rumination is a risk factor that can exacerbate severe depression. The results seem to be in accord with the prior study^30^, showing that negative cognitive styles provide negative inferences for negative life events, but such inferences will be important in contributing to depression when they are recursively activated through rumination.

Self-esteem negatively predicted rumination, and negative life events would impose an impact on depression through the chain mediating roles of self-esteem and rumination. According to the serial multiple mediator model, self-esteem plays as a buffer not only between negative life events and depression, but also between negative life events and rumination. That is to say, self-esteem can make youths less likely to internalize symptoms and reduce symptoms of depression. Moreover, it is considered that high self-esteem positively moderates depression when experiencing negative life events^53^.

In view of the fact that parental divorce is one of the most stressful life events for teenagers^33^, the last hypothesis aimed to examine the moderated effect of parental divorce on the chain mediating model. The results indicated that parental divorce only moderated the path between negative life events and self-esteem. The association between negative life events and self-esteem was not significant among juvenile delinquents whose parents remained married. The possible reason is that teenagers who have an intact family can get more support, attention and love from their parents to lessen the adverse impact of negative life events on self-esteem^54^. Moreover, the youths with parental divorce may face with the divorce-related stigma which may cause them to have a low level of self-esteem, especially most of the participants with parental divorce in our study were from rural areas where the impact of divorce-related stigma is still serious. Hence, juvenile delinquents with non-divorced parents are more likely to have high self-esteem, particularly when they suffer from the same high level of negative life events. Low self-esteem is a vulnerable factor for depression^23^ and parental divorce has an effect on self-esteem. Above all, we can conclude that parental divorce is acted as a risk role that can reduce the self-esteem level and have an indirect impact on depression through self-esteem when juvenile delinquents confront high level of negative life events.

Parental divorce does not moderate the relation between negative life events and depression among Chinese juvenile delinquents. This is partially inconsistent with our initial hypothesis. However, in some ways, the result is reasonable in our study. The relationship between negative life events and depression is not significant after self-esteem and rumination entering into the multiple mediated model. It means that the effects of negative life events on depression are mainly via self-esteem and rumination among juvenile delinquents. The results are consistent with the prior study, which showed that the association between negative life events and depression might be affected by the coping strategies when adolescents encounter stressful life events^55^. Hence, juvenile delinquents with parental divorce or intact family might not make a significant change in the direct impact of negative life events on depression. Another possible reasons is that the subjects in our study are from correctional work-study schools. It suggests that they are exposed with a number of stressful events so that parental divorce is hard to affect depression directly in correctional settings.

### Limitations

The present study has some limitations. First, there were only 30 female participants in this study for they were sampled by using a convenience sampling method and the ratio of girls is 18.1% in all Chinese correctional work-study schools. Therefore, our study did not include the gender variable into the analysis, even some studies found there was a significant gender difference in depression. Second, as participants were sampled from one province in China, maybe it can not fully represent the students in all Chinese correctional work-study schools. The future study should be enlarged sampling range in order to generalize the findings. Third, although the interaction coefficient of negative life events and parental divorce is not significant, it has a negative impact on depression. This may indicate that some factors in the present study could not be involved in, like parental conflict, which may affect the relation between negative life events and depression. Last, the subjects in this study represent one group of juvenile delinquents that stay in correctional work-study schools in China. It is imperative for future research to get a more comprehensive study on the depression of Chinese juvenile delinquents involved other groups, such as the juvenile delinquents in jails.

### Summary

The current study finds that self-esteem and rumination play separate and serial mediating roles between negative life events and depression, and parental divorce plays a moderation role in the association between negative life events and self-esteem. These results can make people have a deeper understanding of the relation between negative life events and depression among juvenile delinquents who study in Chinese correctional work-study schools. Furthermore, there are some implications that may be helpful for concerned staff to design successful treatment and preventative interventions of depression in juvenile delinquents. First, a high level of self-esteem can be a vital protective factor among Chinese juvenile delinquents, especially the group encounter more negative life events than community students. Second, the negative association between negative life events and self-esteem is only significant among juvenile delinquents who experience parental divorce. Hence, it provides a viewpoint for the staff in Chinese correctional work-study schools to pay more attention to juvenile delinquents with parental divorce.

## Data Availability

The data presented in this article are available from the corresponding author upon request.
